# Aspiration of Fractured Tracheostomy Tube in a Prone Positioned COVID-19 Patient: A Case Report and Review of the Literature

**DOI:** 10.4274/TJAR.2023.221167

**Published:** 2023-06-16

**Authors:** Büşra Tezcan, Asiye Yavuz, Bilge Taplamacı Ertuğrul, Abdulaziz Kaplan

**Affiliations:** 1 Clinic of Intensive Care, University of Health Sciences Turkey, Etlik City Hospital, Ankara, Turkey; 2 Clinic of Intensive Care, Ankara City Hospital, Ankara, Turkey; 3 Clinic of Anaesthesiology and Reanimation, Ankara City Hospital, Ankara, Turkey

**Keywords:** Adverse events, critical care, mechanical complications, tracheostomy tube

## Abstract

A 61-year-old male patient diagnosed with Coronavirus disease 2019 (COVID-19) acute respiratory distress syndrome (ARDS) was managed with tracheostomy and intermittent prone positioning in the intensive care unit. After a sudden deterioration, examination of tracheostomy tube (TT) and X-ray of the chest revealed that he had aspirated the fractured TT. The fractured tube was removed through the tracheostomy stoma using a rigid ventilating bronchoscope and forceps. Prone positioning is a beneficial postural therapy capable of improving patient oxygenation. However, it has some complications, like unplanned extubation and facial tissue injury. Percutaneous tracheostomy is also a valuable and safe procedure and has been increasingly performed in critical care patients, including those who suffer from COVID-19 ARDS. Fractures and aspiration of a tracheostomy tube can occur anytime after tracheostomy. We think prone positioning may contribute to the rupture and aspiration of the tracheostomy tube in this study.

**Main Points**

• Percutaneous tracheostomy is usually a beneficial and safe procedure.

• Fractures and aspiration of the tracheostomy tubes are rare but possible complications.

• Tubes can be manufactured as single rather than two connected pieces.

• Mechanical stress associated with prone positioning may have facilitated the fracture of tracheostomy tubes.

## Introduction

Tracheostomy is a standard, reasonable surgical procedure for critically ill patients who require long-term mechanical ventilation.^1^ Due to the increase in demand for critical care arising from the global Coronavirus disease 2019 (COVID-19) pandemic, the number of tracheostomized patients has also generally increased.^2^

Prone positioning is an adjuvant therapy for treating COVID-19-induced acute respiratory distress syndrome (ARDS).^3^ Tracheostomy and prone positioning may reduce morbidity and mortality among mechanically ventilated patients by different mechanisms. Prone positioning relieves external compression forces, recruits the most atelectatic regions of the lungs, and thus recovers ventilation-perfusion ratio mismatching without subjecting the lungs to high airway pressures.^4^ On the other side, tracheostomy improves patient comfort, safety, and communication ability. Better oral and airway care is possible with tracheostomy. At the same time, prone positioning has some airway complications like swelling of the tongue, accidental extubation, and obstruction of the ventilating tube by secretions.^5,6,7,8^ Aspiration of a fractured tracheostomy tube is a rare complication, even in supine-positioned patients.

We present the case of a tracheostomized patient with COVID-19 whose fractured tracheostomy tube dislodged into the left main bronchus.

## Case Presentation

A 61-year-old male patient with no comorbidities was referred to our emergency department for a persistent cough complaint for 6 days. He was positive for COVID-19 (diagnosed using polymerase chain reaction) and was admitted to the intensive care unit (ICU) because of respiratory failure. With worsening respiratory status, he was intubated on the third day of ICU admission without any complications. Due to persistent hypoxemia despite full ventilator support, he was prone at 16 h and supine for 8 h on the following 10 days. Percutaneous tracheostomy using the Griggs forceps-dilational technique was performed on the 14-day of ICU admission. The procedure was uneventful, and a tracheostomy tube (Easyflow; Boen Healthcare Co., Ltd, Jiangsu, China) was inserted easily. Intermittent prone positioning was carried out to optimize oxygenation. There were no acute complications following the procedure.

**Figure 1:**
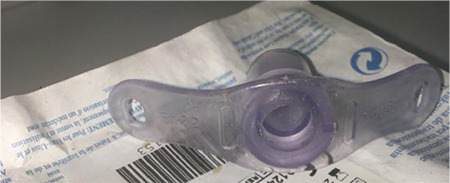
The outer part of the tracheostomy tube.

**Figure 2:**
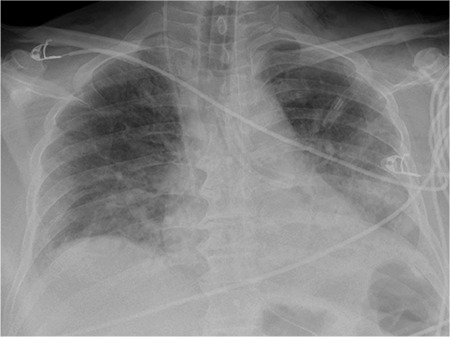
Chest radiograph showing the fractured tracheostomy tube in left main bronchus.

**Figure 3:**
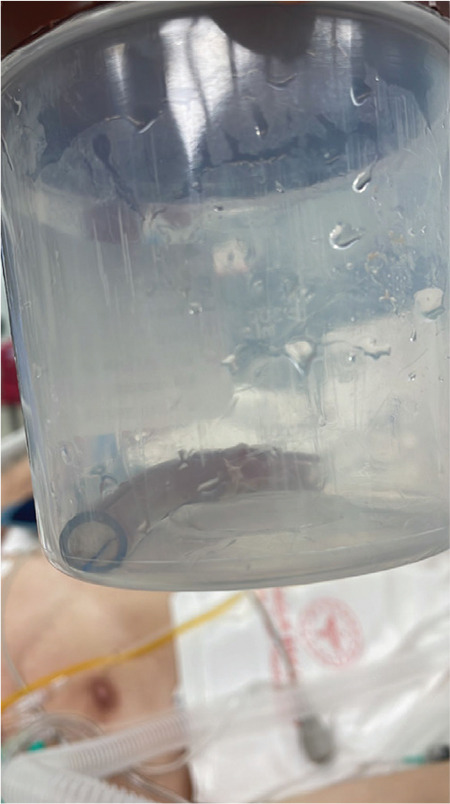
Inner part of the tracheostomy tube removed with bronchoscopy.

However, the patient deteriorated in the prone position five days after the tracheostomy. He developed sudden hypoxia and hypotension and was turned to the prone position. Examination of the tracheostomy tube showed that the flanges were securely tied around the neck while the stem was missing (Figure 1). The patient was orotracheally intubated, and a chest X-ray was performed. It revealed a foreign body in his left main bronchus (RMB) (Figure 2). He was transferred to the operating room for bronchoscopic removal under general anaesthesia. Using a rigid ventilating bronchoscope and forceps, the tube was removed through the tracheostomy stoma (Figure 3). After the procedure, with no complications, he was retransferred to the ICU as orotracheally intubated. The patient died on the 23^rd^ hospital day of multiorgan failure related to septic shock.

## Discussion

Prone positioning of ventilated patients was first used in the 1970s and has been reported as a tool to improve respiratory function in patients with ARDS.^9^ Increased incidence of pressure ulcers, obstruction of endotracheal or tracheostomy tubes, unplanned removals of arterial or venous catheters, unplanned extubation, accidental loss of thoracic or abdominal drains, facial edema, conjunctival hemorrhage, kinking of tubes and catheters, displacement of nasogastric tube and vomiting are some of the complications that have been associated with the use of prone positioning.^10^

Tracheostomy is another ICU practice used for patients requiring an extended mechanical ventilation period. Patients with tracheostomy can be managed in the prone position. Still, since the airway cannot be visualized in this position, the risk of displacement of the tracheostomy tube may be increased.^5,10^ Fracture of the tracheostomy tube with migration into the tracheobronchial tree is a rare complication, even in supine-positioned patients. It is the first report of a fractured tracheostomy tube in a prone-positioned patient.

The first case report of a fractured tracheostomy tube was reported by Howarth^11^ in 1913, although Bassoe and Boe^12^ are known as the first. Since then, this complication has been published in the literature occasionally. Occasionally cases are reported in 65 articles after an extensive literature review.^11,12,13,14,15,16,17,18,19,20,21,22,23,24,25,26,27,28,29,30,31,32,33,34,35,36,37,38,39,40,41,42,43,44,45,46,47,48,49,50,51,52,53,54,55,56,57,58,59,60,61,62,63,64,65,66,67,68,69,70,71,72,73,74,75^ Material and fracture sites of the tubes, possible causes and timing of the events, dislodgement sites, and treatment modalities are some of the topics worth discussing. The fracture of tracheostomy tube can occur from the first minutes of its placement and 22 years later.^53^ Early breakage is usually considered a manufacturing defect.^14^ Fractures after prolonged usage may be due to mechanical (repeated cleaning/boiling or sterilization, suctioning, removal, and reinsertion) or chemical (alkaline bronchial secretions, corrosive cleaning agents) stress.^14,26,28,29,30,35,36,42,56^ Our review of 92 cases revealed that; 66 (71%) of fractures appeared to be associated with prolonged use (repeated boiling, corrosion, and cracking), 13 (14%) appeared to be associated with manufacturing defect and 2 (2%) were attributed to mechanical stress. There are no available data about the rest.

Tracheostomy tubes are made of metal, polyvinyl chloride (PVC), or silicone. Metallic tracheostomy tubes have two main types: Fuller and Jackson. Initially, metallic tubes were thought to allow for prolonged wear due to their physical properties. Silver, steel, copper, or zinc were the materials for manufacturing these tubes, all with poor corrosion resistance to alkaline pH. As a result, they have been corrodible by tracheal secretions and repeated boiling.^12,52,64^ Fractures occur less frequently in PVC and silicone tubes than in metallic tubes.^48^ In this study, the PVC tracheostomy tube was used only for five days before fracturing. Although it is plausible to consider a manufacturing defect that might have contributed to the fracture with its short time use, we believe that prone positioning might also contribute to the mechanical stress created by kinking of the tube. Therefore, this case appears to be an unusual complication of prone positioning.

Most fractures occur at the junction of the cannula and neck plates. As Table 1 reflects the author’s own words, the term “flange” has been used instead of “neck plate” in some reports. On the other hand, the Fuller metallic tubes have flanges at the distal end of the main cannulas and sometimes get fractured at the junction of these flanges.^53^ There are 31 reports about fractured PVC tubes in the literature; two have no data about the fracture sites, and only one siliconized PVC tube was fractured from the mid-shaft. In our case, the tracheostomy tube fracture occurred at the junction of the cannula and neck plate, similar to the other reported cases of fractured PVC tracheostomy tubes. The manufacturers of PVC tubes should be warned about strengthening the connection between the two components of the tubes. Li et al.^42^ and friends mentioned that they filed a Medical Device Alert form, and the tube was returned to the supplier in their report. Hence, the supplier redesigned to incorporate a new shaft-to-head base assembly method to strengthen the connection.

RMB is more exposed to the lodgment of foreign bodies since it is mostly vertically positioned and has a larger diameter than the left main bronchus.^75^ It was also the most common dislodgement site for fractured tubes (37 cases).

Clinical presentation depends on factors such as patient status, dislodgement style, and site of the fractured tube. Patients tracheostomized for chronic respiratory disorders can present with mild respiratory distress, cough, wheezing, recurrent pneumonia, and difficulty suctioning or reinserting the inner tube.^64,74^ Some cases even remain asymptomatic in which the fractured part acts like a stent in the trachea or main bronchus.^36,62^ Death may also occur, especially in pediatric patients, probably due to the small airway caliber.^28,54^ Our patients suffered from acute and severe ARDS, and disconnection of the two parts of the tube resulted in inadequate mechanical ventilation. He needed urgent orotracheal intubation because of sudden hypoxia.

Large foreign bodies in the tracheobronchial tree are usually removed by rigid bronchoscopy. It is also recommended for the removal of fractured tracheostomy tubes in the literature. A bronchoscope is usually inserted through the tracheostomy stoma to avoid vocal cords and oral cavity from mechanical injury caused by a fractured tube during removal.^64^ Flexible bronchoscopy, local exploration of the tracheostome, and removal with forceps, nasal endoscope, or Desjardin’s forceps under C-arm guidance through the tracheostome, thoracotomy, and bronchotomy are some other treatment approaches.^33,40,61,63,65^

## Conclusion

Fracture and aspiration of the tracheostomy tube is a rare complication that can occur anytime after tracheostomy. Regular care and replacement of worn-out tracheostomy tubes are essential to avoid this complication in patients with chronic tracheostomy. We also recommend checking for manufacturing defects before insertion. Tubes can be manufactured as single rather than two connected pieces. On the other hand, we think that mechanical stress associated with prone positioning may have facilitated the fracture of the tracheostomy tube in this study. Tracheostomy tubes should be avoided kinking and mechanical stress during prone positioning.

## Figures and Tables

**Table 1:**
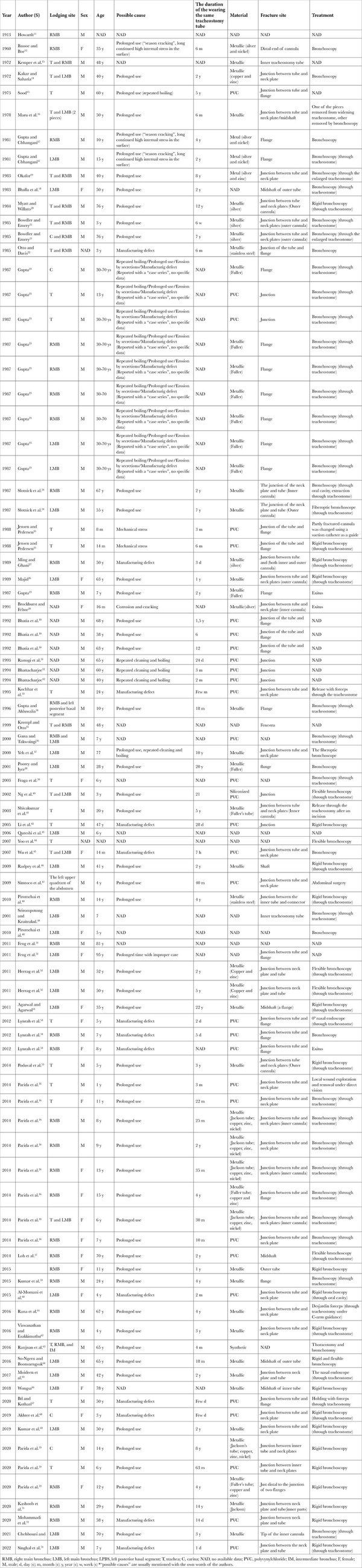
Summary of the previous case reports

## References

[1] Stauffer JL, Olson DE, Petty TL (1981). Complications and consequences of endotracheal intubation and tracheotomy. A prospective study of 150 critically ill adult patients. Am J Med..

[2] Williams T, McGrath BA (2021). Tracheostomy for COVID-19: evolving best practice. Crit Care..

[3] Ghelichkhani P, Esmaeili M (2020). Prone Position in Management of COVID-19 Patients; a Commentary. Arch Acad Emerg Med..

[4] Curley MA, Hibberd PL, Fineman LD, et al (2005). Effect of prone positioning on clinical outcomes in children with acute lung injury: a randomized controlled trial. JAMA..

[5] Durbin CG Jr (2005). Indications for and timing of tracheostomy. Respir Care..

[6] Pivalizza EG, Katz J, Singh S, Liu W, McGraw-Wall BL (1998). Massive macroglossia after posterior fossa surgery in the prone position. J Neurosurg Anesthesiol..

[7] Dingeman RS, Goumnerova LC, Goobie SM (2005). The use of a laryngeal mask airway for emergent airway management in a prone child. Anesth Analg..

[8] Lin JA, Wong CS, Cherng CH (2005). Unexpected blood clot-induced acute airway obstruction in a patient with inactive pulmonary tuberculosis during lumbar spine surgery in the prone position--a case report. Acta Anaesthesiol Taiwan..

[9] Bryan AC (1974). Conference on the scientific basis of respiratory therapy. Pulmonary physiotherapy in the pediatric age group. Comments of a devil’s advocate. Am Rev Respir Dis.

[10] Dirkes S, Dickinson S, Havey R, O’brien D (2012). Prone positioning: is it safe and effective?. Crit Care Nurs Q.

[11] Howarth W (1913). Piece of Broken Tracheotomy Tube removed from the Right Bronchus. Proc R Soc Med.

[12] Bassoe HH, Boe J (1960). Broken tracheotomy tube as a foreign body. Lancet..

[13] Kemper BI, Rosica N, Myers EN, Sparkman T (1972). Inner migration of the inner cannula: an unusual foreign body. Eye Ear Nose Throat Mon..

[14] Kakar PK, Saharia PS (1972). An unusual foreign body in the tracheo-bronchial tree. J Laryngol Otol..

[15] Sood RK (1973). Fractured tracheostomy tube. J Laryngol Otol..

[16] Maru YK, Puri ND, Majid A (1978). An unusual foreign body in the tracheobronchial tree. J Laryngol Otol..

[17] Gupta AK, Chhangani DL (1981). Fractured tracheostomy tube. Indian J Otolaryngol..

[18] Bhalla K, Bais AS, Mahindra S (1983). An unusual bronchial foreign body. Indian J Otolaryngol..

[19] Okafor BC (1983). Fracture of tracheostomy tubes. Pathogenesis and prevention. J Laryngol Otol..

[20] Myatt JK, Willatts DG (1984). An inhaled tracheostomy tube. Successful anaesthetic management. Anaesthesia..

[21] Bowdler DA, Emery PJ (1985). Tracheostomy tube fatigue. An unusual cause of inhaled foreign body. J Laryngol Otol..

[22] Otto RA, Davis W (1985). Tracheostomy tube fracture: an unusual etiology of upper respiratory airway obstruction. Laryngoscope..

[23] Gupta SC (1987). Fractured tracheostomy tubes in the tracheo-bronchial tree: (a report of nine cases). J Laryngol Otol..

[24] Slotnick DB, Urken ML, Sacks SH, Lawson W (1987). Fracture, separation, and aspiration of tracheostomy tubes: management with a new technique. Otolaryngol Head Neck Surg..

[25] Jensen OV, Pedersen U (1988). Fractures in polyvinyl chloride tracheostomy tubes. J Laryngol Otol..

[26] Majid AA (1989). Fractured silver tracheostomy tube: a case report and literature review. Singapore Med J..

[27] Ming CC, Ghani SA (1989). Fractured tracheostomy tube in the tracheobronchial tree. J Laryngol Otol..

[28] Brockhurst PJ, Feltoe CK (1991). Corrosion and fracture of a silver tracheostomy tube. J Laryngol Otol..

[29] Gupta SC (1991). Tracheostomy tube fracture an unusual fatal complication of tracheostomy. Indian J Otolaryngol..

[30] Bhatia S, Malik MK, Bhatia BP (1992). Fracture of tracheostomy tubes--report of 3 cases. Indian J Chest Dis Allied Sci..

[31] Rastogi N, Datta NR, Ayyagagi S (1993). Fractured polyvinyl chloride tracheostomy tube as a foreign body in tracheobronchial tree. Indian J Chest Dis Allied Sci..

[32] Bhattacharjee N (1994). Fractured tracheostomy tubes: 3 case reports. Bangladesh Med Res Counc Bull..

[33] Kochhar LK, Chaudhry S, Sikand A, Kumar A (1995). Fractured tracheostomy tubes: a per-operative emergency. Med J Armed Forces India..

[34] Gupta SC, Ahluwalia H (1996). Fractured tracheostomy tube: an overlooked foreign body. J Laryngol Otol..

[35] Krempl GA, Otto RA (1999). Fracture at fenestration of synthetic tracheostomy tube resulting in a tracheobronchial airway foreign body. South Med J..

[36] Gana PN, Takwoingi YM (2000). Fractured tracheostomy tubes in the tracheobronchial tree of a child. Int J Pediatr Otorhinolaryngol..

[37] Yeh PS, Hsu YL, Kuo PS (2000). Aspiration of a broken metallic tracheostomy tube: An unusual cause of tracheobronchial foreign body. Thorac Med..

[38] Poorey VK, Iyer A (2001). Unusual foreign body (broken tracheostomy tube) in left main bronchus. Indian J Otolaryngol Head Neck Surg..

[39] Fraga JC, Pires AF, Komlos M, Takamatu EE, Camargo LG, Contelli FH (2003). Remoção de corpo estranho da via aérea de criança por broncoscopia através de traqueotomia ou traqueostomia [Bronchoscopic removal of foreign body from airway through tracheotomy or tracheostomy]. J Pediatr (Rio J)..

[40] Ng DK, Cherk SW, Law AK (2002). Flexible fiberoptic bronchoscopic removal of a fractured synthetic tracheostomy tube in a 3-year-old child. Pediatr Pulmonol..

[41] Shivakumar AM, Naik AS, Prashanth KB, Yeli SS, Yogesh BS (2003). Unusual foreign body in the trachea. Indian J Otolaryngol Head Neck Surg..

[42] Li AM, Angadi PS, Iossifidis F (2005). Failure of a dual-cannula tracheostomy tube. Anaesthesia..

[43] Qureshi SS, Chaukar D, Dcruz A (2006). Fractured tracheostomy tube in the tracheo-bronchial tree. J Coll Physicians Surg Pak..

[44] Yoo JC, Chang MY, Jung YH, Jin HR (2007). Tracheal foreign body by accidental fracture of tracheostomy tube. Korean Journal of Bronchoesophagology..

[45] Wu CT, Lin JJ, Yeh R (2007). Migration of fragmented tracheostomy tube into left main bronchus. International Journal of Pediatric Otorhinolaryngology Extra..

[46] Radpey BAZ, Pezhan S, Dabir SH, Parsa T, Radpey MZ (2009). Fracture and aspiration of tracheostomy tube. Tanaffos..

[47] Simtoco MJD, Soriano-Castaneda S, Alonzo DM, Reyes-Quintos MRT (2009). Fractured Tracheostomy Tube Ingestion in a Pediatric Patient. Philippine Journal of Otolaryngology-Head and Neck Surgery..

[48] Piromchai P, Lertchanaruengrit P, Vatanasapt P, Ratanaanekchai T, Thanaviratananich S (2010). Fractured metallic tracheostomy tube in a child: a case report and review of the literature. J Med Case Rep..

[49] Srirompotong S, Kraitrakul S (2001). Fractured inner tracheostomy tube : An unusual tracheobronchial foreign body. Srinagarind Med J..

[50] Gupta SC (1991). Tracheostomy tube fracture an unusual fatal complication of tracheostomy. Indian J Otolaryngol.

[51] Feng CC, Sun KC, Liang TY, Tu MY, Woo PT (2011). A fractured tracheobronchial suction catheter and fractured tracheostomy tube in the tracheobronchial tree: two case reports. J Emerg Crit Care Med..

[52] Herrag M, Sajiai H, Rochdi Y, et al (2011). Flexible bronchoscopic removal of a fractured metallic tracheostomy tube. J Bronchology Interv Pulmonol..

[53] Agarwal N, Agarwal R (2011). Fractured tracheostomy tube migrating into the tracheobronchial tree: a rare complication. Indian J Chest Dis Allied Sci..

[54] Lynrah ZA, Goyal S, Goyal A, Lyngdoh NM, Shunyu NB, Baruah B, Dass R, Yunus M, Bhattacharyya P (2012). Fractured tracheostomy tube as foreign body bronchus: our experience with three cases. Int J Pediatr Otorhinolaryngol.

[55] Poduval J, Benazir F, Ninan P (2014). Pneumopericardium - an unusual complication of broken tracheostomy tube presenting as foreign body trachea. J Laryngol Voice..

[56] Parida PK, Kalaiarasi R, Gopalakrishnan S, Saxena SK (2014). Fractured and migrated tracheostomy tube in the tracheobronchial tree. Int J Pediatr Otorhinolaryngol..

[57] Loh TL, Chin R, Flynn P, Jayachandra S (2014). Fracture and aspiration of a tracheostomy tube. BMJ Case Rep..

[58] Guggarigoudar L (2015). Recurrent fracture of outer metallictracheostomy tube into right main bronchus. Int J Pharm Bio Sci..

[59] Kumar S Jha, Jagadheesh JSB, Thirunavukarasu M (2015). Broken tracheotomy tube: a case report. Otolaryngology Online Journal..

[60] Al-Momani HM, Alzaben KR, Mismar A (2015). Upper airway obstruction by a fragmented tracheostomy tube: Case report and review of the literature. Int J Surg Case Rep.

[61] Rana I, Chongtham C, Kumar JM (2016). Retrieval of fractured metallic tracheostomy tube - An innovative approach. Annals of International Medical and Dental Research..

[62] Viswanathan A, Esakkimuthu S (2016). When a safety-valve became a ticking time-bomb: fractured tracheostomy tube as a tracheobronchial foreign body in a child. IJPMR..

[63] Ranjnan K, Phookan J, Devi HR, Das MR (2016). Broken synthetic tracheostomy tube presenting as tracheobronchial foreign body-a case report. Journal of Dental and Medical Sciences..

[64] So-Ngern A, Boonsarngsuk V (2016). Fractured metallic tracheostomy tube: A rare complication of tracheostomy. Respir Med Case Rep..

[65] Moideen SP, Arun G, Mohan M, Afroze KH (2017). Fractured tracheostomy tubean unusual foreign body in tracheobronchial tree. Bengal Journal of Otolaryngology and Head Neck Surgery..

[66] Wongsa P (2018). Anesthetic management for a patient with fractured silver tracheostomy tube in the left main bronchus undergoing rigid bronchoscopy. Thai Journal of Anesthesiology..

[67] Bd V, Kothari N (2020). Use of Pilot Balloon to Fish Out Fractured Tracheostomy Tube: A Case Report. A A Pract..

[68] Akhter T, Khan T, Genie F (2019). Broken tracheostomy tube presenting as tracheobronchial foreign body: a rare case report. World Journal of Pharmaceutical Research..

[69] Kumar S, Singh HP, Hajela A (2019). Lifesaving device presenting as bronchial foreign body. Int J Otorhinolaryngol Clin..

[70] Parida PK, Kalaiarasi R, Alexander A, Saxena SK (2020). Factors Associated with Fracture and Migration of Tracheostomy Tube into Trachea in Children: A Case Series. Iran J Otorhinolaryngol..

[71] Kashoob M, Al Washahi M, Tandon R (2020). Aspiration Pneumonia Due to Migration of Fracture Tracheostomy Tube after 14 Years of Use. Oman Med J..

[72] Mohammadi M, Sasaa MAZ, Alomaran AZ, Alhamaidah MF, Spoor J, Roomi AB (2020). Case report of a rare complication of tracheostomy tube in intensive care unit. International Journal of Pharmaceutical Research..

[73] Chehbouni M, Benhoummad O (2021). Acute Respiratory Distress Revealing an Unrecognized Tracheostomy Cannula at the Bronchial Level in the Pandemic COVID Era. EJMED.

[74] Singhal G, Kumar P, Goel S, Goit S, Pathak A (2022). A case of migration of fractured tracheostomy tube—a case presentation. Egypt J Otolaryngol..

[75] Saki S, Norouzi S (2020). Unusual presentation of foreign body aspiration in adult. J Adv Pharm Edu..

